# Extracellular Hsp90α Supports the ePKM2-GRP78-AKT Axis to Promote Tumor Metastasis

**DOI:** 10.3389/fonc.2022.906080

**Published:** 2022-06-30

**Authors:** Shaosen Zhang, Caihong Wang, Jiujun Ju, Caixia Wang

**Affiliations:** ^1^ Department of Etiology and Carcinogenesis, National Cancer Center/Cancer Hospital, Chinese Academy of Medical Sciences and Peking Union Medical College, Beijing, China; ^2^ Beijing Friendship Hospital, Capital Medical University, Beijing, China; ^3^ Yantai Key Laboratory of Pharmacology of Traditional Chinese Medicine in Tumor Metabolism, School of Integrated Traditional Chinese and Western Medicine, Binzhou Medical University, Yantai, China

**Keywords:** PKM2, extracellular interacting network, eHsp90α, metastasis, lung cancer

## Abstract

Tumor-secreted proteins can provide numerous molecular targets for cancer diagnosis and treatment. Of note, pyruvate kinase M2 (PKM2) is secreted by tumor cells to promote malignant progression, while its regulatory mechanism or the interacting network remains uncovered. In the present study, we identified extracellular heat shock protein 90 alpha (eHsp90α) as one potential interacting protein of ePKM2 by mass spectrometry (MS), which was further verified by pull-down and co-immunoprecipitation analysis. Later, we found that eHsp90α enhanced the effect of ePKM2 on migration and invasion of lung cancer cells. Blocking of Hsp90α activity, on the other hand, attenuated tumor migration or invasion induced by ePKM2. Eventually, the *in vivo* role of Hsp90α in regulating ePKM2 activity was validated by the mouse xenograft tumor model. Mechanistically, we found that eHsp90α binds to and stabilizes ePKM2 to protect it from degradation in the extracellular environment. Besides, eHsp90α promoted the interaction of ePKM2 with cell surface receptor GRP78, which leads to the activation of the ePKM2/GRP78/AKT axis. Collectively, we unraveled the novel molecular mechanism of eHsp90α in regulating ePKM2 activity during tumor progression, which is beneficial for the development of new treatments against lung cancer.

## Introduction

Tumor cells secrete a series of molecules involved in the cross talk between themselves and adjacent stromal cells in the tumor microenvironment (TME) (1). Of note, tumor-secreted proteins are known to be involved in tumor initiation, progression, and relapse following treatment, which highlights the role of tumor-secreted proteins as biomarkers or drug targets in tumor diagnosis/treatment (2, 3). Extracellular proteins, such as matrix metalloproteinases (MMPs) (4), vascular endothelial growth factor (VEGF) (5), and Hsp90α (6, 7) have been reported to promote cell motility and angiogenesis in TME. Although recent discoveries have broadened our knowledge of tumor-associated proteins, the interaction network or the molecular mechanism of extracellular proteins acting on tumor progression remains unclear.

As rate-limiting enzymes in cellular glycolysis, pyruvate kinase has four isoforms (PKM1, PKM2, PKL, and PKR) (8). Among them, pyruvate kinase M2 (PKM2) is predominantly expressed in the fetus, while pyruvate kinase M1 (PKM1) can replace PKM2 during the process of development (9). Different from PKM1, PKM2 was also preferentially expressed in cancer cells (9). Previous works had demonstrated that PKM2 could convert phosphoenolpyruvate (PEP) to pyruvate during the final step of glycolysis, which was crucial for aerobic glycolysis in cancer cells (10). PKM2 was found highly expressed in invasive cancer cells compared with normal cells (11), and it was reported that the expression of PKM2 was reversely correlated with survival rate in patients (12). On the cellular level, PKM2 is preferably localized in the cytosol, nucleus, and mitochondria. In cancer cells, dimeric PKM2 in the cytosol has low enzymic activity, which leads to the accumulation of glycolytic intermediates and facilitates cell proliferation (13). Besides that, PKM2 could also act as a protein kinase (14–17) and transcriptional regulator (18). For example, PKM2 was reported to work as a regulatory component of transcription factor HIF-1α in the nucleus (19). In mitochondria, it is revealed that PKM2 interacted with Bcl2 to prevent Cul3-based E3 ligase binding to Bcl2 (14).

Extracellular PKM2 (ePKM2) is widely expressed in lung cancer, gastrointestinal cancer, ovarian cancer, pancreatic ductal adenocarcinoma, and renal cell carcinoma patients (20). Li et al. reported that secreted PKM2 could facilitate tumor growth (21). Extracellular PKM2 was also found to facilitate the migration of colon cancer cells (22, 23). Recently, Han et al. reported that extracellular PKM2 can facilitate fibrosis progression (24). Generally, ePKM2 can interact with other proteins to activate multiple downstream signals. Our previous study revealed that ePKM2 binds to the extracellular domain of integrin β1 to activate the FAK/ERK signaling pathway (25). In the circulation system, secreted PKM2 was also found to activate PI3K/Akt and Wnt/β-catenin pathways (21). Moreover, exosomal PKM2 exacerbates cisplatin resistance in NSCLC cells (26), and ectosomal PKM2 promotes cancer progression by remodeling TME (27). However, it is little known about the existence of any other extracellular factors involved in ePKM2-mediated tumor progression, and the potential function of extracellular PKM2 has not been elaborated yet.

Here, we investigated the regulatory mechanism of ePKM2 by exploring the extracellular interacting partners of ePKM2. We discovered Hsp90α as a novel binding partner of ePKM2, which facilitates ePKM2-induced tumor metastasis by increasing its stability in the extracellular environment. Moreover, we found that the GRP78/AKT axis mediates ePKM2-initiated tumor migration and invasion. Overall, our study revealed a previously unidentified molecular mechanism of Hsp90α in regulating ePKM2 activity and provides new extracellular targets for lung cancer treatment.

## Materials and methods

### Cell Culture

The human lung cancer A549 cell line and H1299 cell line were both obtained from China Infrastructure of Cell Line Resources (Beijing, China). Dulbecco’s modified Eagle’s medium (DMEM) or RPMI-1640 (Wisent, Nanjing, China) supplemented with 10% fetal bovine serum (FBS) was used to maintain the growth of cells. The glucose in DMEM media for the A549 cell culture is 4,500 mg/l. The glucose in RPMI-1640 media for the H1299 cell culture is 2,000 mg/l. All cells were maintained cultivated at 37°C in a humidified incubator containing 5% CO_2_. When the cell density reached 80%, the media were aspirated. Cells were washed two times with PBS and three times with serum-free media. Serum-free media were added to maintain the cell growth for 12 h. Then the conditioned media were collected and concentrated by centrifugation.

### Cell Line Construction and Protein Purification

The GRP78 stably knocked-down cell lines and control cell lines were generated by lentivirus infection. Briefly, the packaging vector (psPAX2), envelope plasmid (pVSVG), and transfer plasmid (pLKO.1) were co-transfected to HEK293T cells. After 72 h, we collected the HEK293T medium containing the virus. The 0.45-μM PVDF filter was used to filter the virus. After that, the virus was transferred to A549 cells. Puromycin (500 ng/ml) was used to select the positive cells. The shRNA sequences used in this paper are listed in **Table S2**.

Human full-length PKM2 and Hsp90α with an N-terminal His tag were cloned into the plenty-CMV-BSD vector, which was transfected with plasmid psPAX2 and pVSVG together into HEK293T cells as above. The cells exogenously expressing PKM2 and Hsp90α were washed with PBS three times and cultured in serum-free DMEM and RPMI-1640 media for 12 h. Then, the conditioned media were collected and concentrated by centrifugation. The extracellular PKM2 and Hsp90α were purified by Ni-NTA affinity chromatography.

On the other hand, human full-length PKM2 and Hsp90α cDNA were amplified by polymerase chain reaction (PCR). The PCR products were cloned into the pQE-80L empty vector. His-tag was added to the N-terminal of PKM2 and Hsp90α. rHsp90α and rPKM2 proteins with the N-terminal tag were produced in *Escherichia coli* and purified by Ni-NTA affinity chromatography and ion exchange column.

### Pull-Down Assay

Cyanogen bromide-activated matrices (CNBr) (Sigma-Aldrich, St. Louis, MO) were used to covalently couple the His-tagged rPKM2 protein. The CNBr-coupled rPKM2 was incubated with A549-conditioned medium or crude fractions of the plasma membrane of A549 cells. Samples were washed three times with Tris buffer (50 mM Tris, 150 mM NaCl, 0.5% NP40), then analyzed by immunoblot.

### Mass Spectrometry Analysis

The whole-gel slices containing protein bands were excised and digested by sequencing grade modified trypsin. Liquid chromatography–mass spectrometry (Agilent 6300 Series Ion Trap Liquid Chromatography/Mass Systems) was used to analyze the peptide mixture. The Swiss-Prot database was used to pilot mass data. All annotations were extracted from the UniProt database.

### Co-Immunoprecipitation Assay

Conditioned media from A549 and H1299 cells were collected and incubated with anti-Hsp90α antibody (Abcam, Cambridge, MA, USA, ab2928, 1:2,000 dilution), anti-GRP78 antibody (Santa Cruz, Dallas, TX, USA, SC-1050, 1:1,000 dilution), and protein G-Sepharose (Roche, Basel, Switzerland) overnight at 4°C with constant rotation. Before analyzing by immunoblot, we washed immuno-complexes three times with IP lysis buffer. Antibodies against PKM2 (#4053, 1:1,000 dilution) and β-actin (#3700, 1:1,000 dilution) were obtained from Cell Signaling Technologies (Danvers, MA).

### Wound Healing Assay

The cells were plated in six-well plates. To produce a wound, the monolayer cells were scraped in a straight line using a 10-μl pipette tip. PBS was added to wash the plate and remove the detached cells. Then serum-free medium was added to incubate the cell. A Nikon inverted microscope was used to obtain photographs of the scratch after 24 and 48 h of wounding. We used Image-Pro Plus 6.0 software to analyze gap width. The average of three independent experiments was shown in the text.

### Cell Invasion Assay

Cells were treated with rPKM2, rHsp90α, and Hsp90α antibodies at 37°C for 30 min, respectively. Then the pretreated cells were seeded into the upper Matrigel (Corning, New York, USA)-coated chamber of Millicell culture inserts (8 μm; Merk Millipore, Darmstadt, Germany). The lower chamber was added with the culture medium containing reagents. After 12–24 h incubation, the cells were fixed with 4% polyformaldehyde. Then the upper cells were swabbed and crystal violet was used to identify the migrated cells. The Nikon inverted microscope was used to obtain photographs.

### Xenograft Studies

BALB/c nude female mice were housed in pathogen-free barrier facilities. Moreover, the mice were maintained in a 12-h light/dark cycle at 22–26°C with sterile pellet food and water supply. All mice were divided into a maximum of six mice per cage. The laboratory animal facility has been accredited by AAALAC (Association for Assessment and Accreditation of Laboratory Animal Care International) and the IACUC (Institutional Animal Care and Use Committee). Binzhou Medical University approved all procedures in this study by the Guide for the Care and Use of Animals. For the subcutaneous xenograft model, we collected A549 cells with a concentration of 1 × 10^6^ cells/ml and subcutaneously injected 0.1 ml of PBS into female nude mice (n = 7-8 per group). A caliper was used to measure the volume of mice twice per week. Recombinant PKM2 and Hsp90α were injected with 100 mg/mouse. Moreover, Hsp90α antibodies were injected with 80 mg/mouse. All reagents were injected twice per week *via* a tail vein. After about 40 days, we isolated and weighed tumors and the sentinel lymph nodes. For the tail vein mouse model, A549 cells with 1 × 10^6^ cells/ml were collected and suspended in 0.1 ml of PBS. We injected A549 cells into the tail vein of 8-week-old nude mice (nine mice per group). After about 2 months of injection, all mice were killed. The lung and liver were isolated and embedded with paraffin. H&E was used to stain the tissues to identify metastatic deposits.

### Statistical Analysis

Data are presented as means ± SD, or means ± SEM. Comparisons between two groups were performed by two-tailed, unpaired Student’s *t*-test by GraphPad Prism (GraphPad Software, USA). The repeated measures of tumor volume were analyzed by longitudinal models. All *P* values less than 0.05 were considered as statistically significant differences.

## Results

### Hsp90α Is an Extracellular Interacting Protein of PKM2

**Figure 1:**
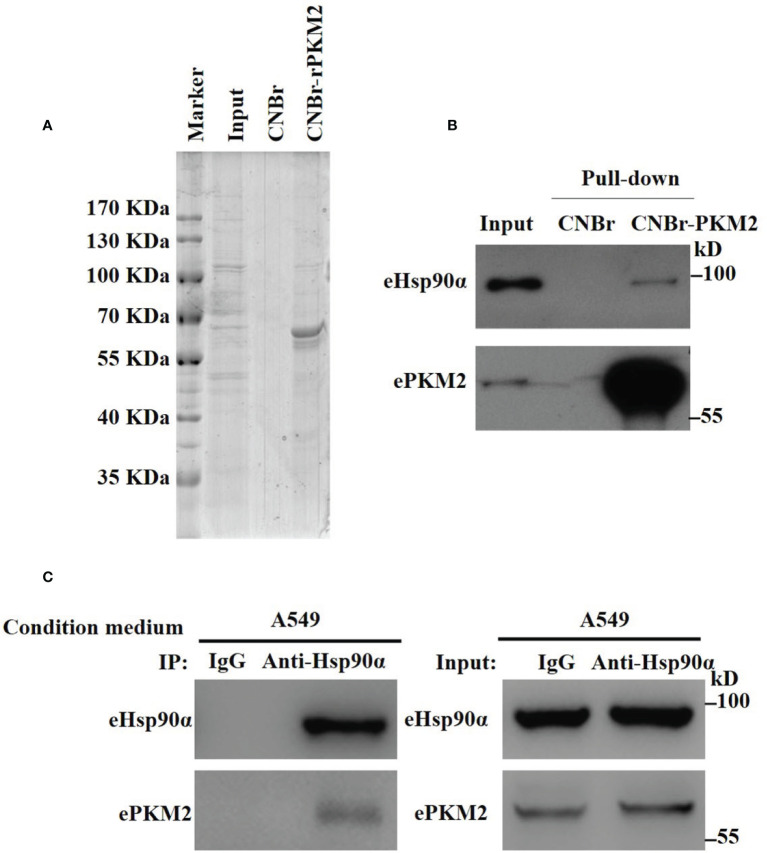
Identification of eHsp90α as an extracellular interacting protein of PKM2. **(A)** Eluted fraction of CNBr or CNBr-rPKM2 affinity chromatography was applied to SDS-PAGE before mass spectrometry analysis. **(B)** Pull-down assay showing the interaction between extracellular PKM2 and Hsp90α in A549-conditioned media. **(C)** A549-conditioned media was collected and immunoprecipitated with IgG or anti-Hsp90α antibodies. The immunoprecipitated samples were subjected to WB analysis with the indicated antibodies to detect the interaction between extracellular PKM2 and Hsp90α.

To explore extracellular binding partners of ePKM2, we performed a pull-down assay of conditioned media from lung cancer cell line A549. Specifically, recombinant PKM2 (rPKM2) was conjugated with cyanogen bromide-activated Sepharose beads and was incubated with the conditioned media from A549 cells. The precipitated fractions were resolved by SDS-PAGE (**Figure 1A**). Subsequently, mass spectrometry was used to identify PKM2-interacting extracellular proteins. Proteins with top 10 scorings were listed (**Table S1**), of which Hsp90α has the highest score. As an extracellular chaperone, eHsp90α has been reported to work with other extracellular effectors to promote tumor metastasis (7, 28). Therefore, we hypothesized the role of eHsp90α in regulating signals downstream of ePKM2.

To explore the biological function of eHsp90α on ePKM2’s activity, we first detected the physical interaction between eHsp90α and ePKM2. The pull-down assay result reflected the interaction between eHsp90α and ePKM2 (**Figure 1B**). To verify this, A549- and H1299-conditioned media were collected for co-immunoprecipitation (Co-IP) analysis, and it revealed that ePKM2 interacts with eHsp90α in the extracellular environment (**Figure 1C**, **Figure S1**).

### ePKM2 and eHsp90α Synergistically Promote Cancer Cell Migration and Invasion

**Figure 2:**
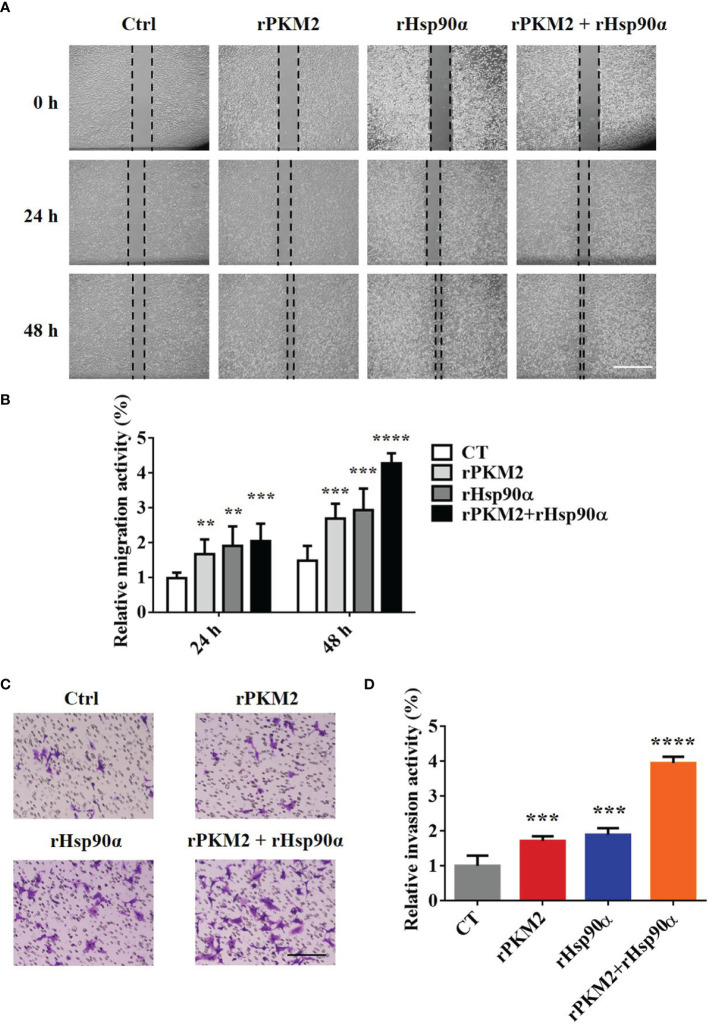
eHsp90α enhances the promoting effects of ePKM2 on tumor migration and invasion. **(A)** Representative images of A549 cell migration ability after treatment with rPKM2 (5 μg/ml), rHsp90α (5 μg/ml), or the combination of rPKM2 and rHsp90α. Scale bar, 200 μm. **(B)** Quantitative results of A549 cell migration ability after treatment with rPKM2 (5 μg/ml), rHsp90α (5 μg/ml), or the combination of rPKM2 and rHsp90α. **(C)** Representative images of A549 cell invasion ability after the treatment with rPKM2 (5 μg/ml), rHsp90α (5 μg/ml), or the combination of rPKM2 and rHsp90α. Scale bar, 200 μm. **(D)** Quantitative results of A549 cell invasion ability after the treatment with rPKM2 (5 μg/ml), rHsp90α (5 μg/ml), or the combination of rPKM2 and rHsp90α. Data are represented as mean ± SD. NS, not significant; **p < 0.01, ***p < 0.001, ****p < 0.0001, two-tailed Student’s *t* tests; n = 3 biological replicates.

It is revealed by our previous study that the secreted PKM2 could promote lung cancer progression (25). Therefore, it is worthwhile to investigate the biological role of interaction between Hsp90α and PKM2 in ePKM2-dependent tumor migration and invasion. To address this, we performed wound healing and transwell assays by treating A549 cells with rPKM2 and rHsp90α. As shown in **Figures 2A, B**, rHsp90α and rPKM2 can both facilitate tumor cell migration, which was consistent with our previous findings (25, 29). Furthermore, coadministration of rPKM2 (5 μg/ml) and rHsp90α (5 μg/ml) was used to investigate whether eHsp90α is involved in ePKM2-promoted tumor cell migration. Our results showed that rHsp90α elevates rPKM2-promoted cell migration. Consistently, tumor cell invasion was also increased following coadministration of rPKM2 and rHsp90α (**Figures 2C, D**). These results demonstrated that ePKM2 and eHsp90α work synergistically to promote cancer cell migration and invasion.

### ePKM2 Promotes Tumor Cell Migration and Invasion Dependent on eHsp90α

**Figure 3:**
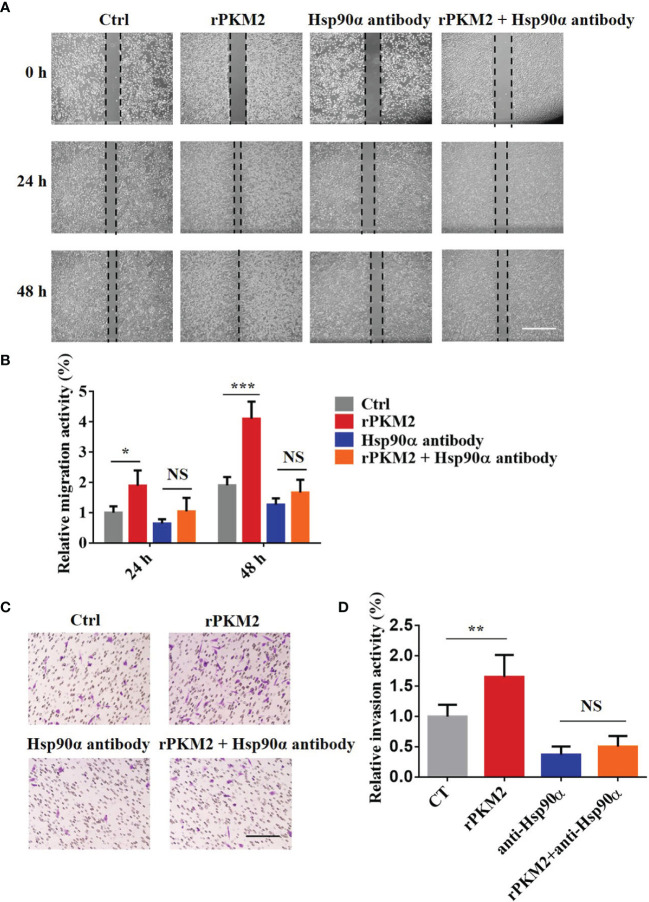
Extracellular PKM2 promotes tumor migration and invasion dependent on extracellular Hsp90α. **(A)** Representative images of A549 migration assay (scale bar, 200 μm) with PBS, rPKM2, anti-Hsp90α, or anti-Hsp90α and rPKM2 together. **(B)** Quantified results of A549 migration assay with PBS, rPKM2, anti-Hsp90α, or anti-Hsp90α and rPKM2 together. **(C)** Representative images of A549 invasion assay (scale bar, 200 μm) with PBS, rPKM2, anti-Hsp90α, or anti-Hsp90α and rPKM2 together. **(D)** Quantified results of A549 invasion assay with PBS, rPKM2, anti-Hsp90α, or anti-Hsp90α and rPKM2 together. Data are represented as mean ± SD. NS, not significant; *p < 0.05; **p < 0.01, ***p < 0.001, two-tailed Student’s *t* tests; n = 3 biological replicates.

It has been reported that Hsp90α, as a molecular chaperone, has essential functions in various biological processes (30). To elucidate how eHsp90α influences ePKM2-promoted tumor cell migration and invasion, we treated A549 cells with rPKM2 and anti-Hsp90α antibodies simultaneously. As shown in **Figures 3A, B**, rPKM2 enhanced tumor cell migration, which was dramatically attenuated with the treatment of Hsp90α antibody. Consistent with that, rPKM2-promoted tumor cell invasion was also obviously restrained after treatment with anti-Hsp90α antibody (**Figures 3C, D**). To further investigate the function of eHsp90α on ePKM2-promoted tumor cell migration and invasion, similar assays were performed on H1299 cells. As expected, rPKM2-promoted tumor cell migration and invasion were also obviously restrained after treatment with anti-Hsp90α antibody (**Figures S2A–D**). Collectively, these results demonstrated that ePKM2 promotes tumor cell migration and invasion which is regulated by eHsp90α.

### Hsp90α Influences ePKM2-Promoted Tumor Progression *In Vivo*

**Figure 4:**
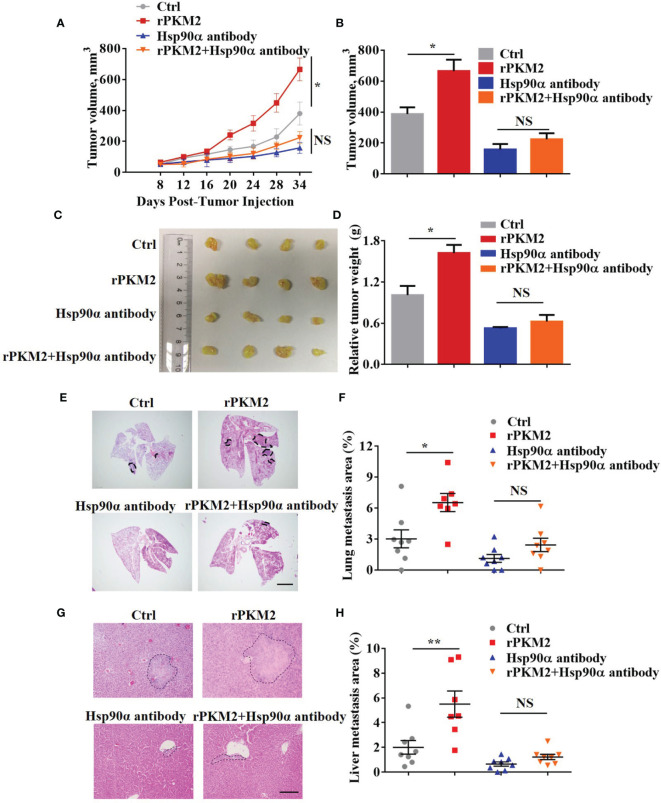
Extracellular PKM2 promotes tumor progression dependent on extracellular Hsp90α. **(A–D)** Tumor progression generated by subcutaneous injection of A549 cells into nude mice (n = 7–8/group). Mice were treated twice per week with PBS, rPKM2, anti-Hsp90α, or anti-Hsp90α and rPKM2 together by tail vein injection. **(A)** Tumor growth curves of A549 tumor xenografts. **(B)** Tumor volume at last time point. **(C)** The gross observation of tumors. **(D)** Tumor weight of A549 tumor xenografts. **(E–H)** Tumor metastasis generated by tail vein injection of A549 cells into nude mice (n = 7–8/group). Mice were treated twice per week with PBS, rPKM2, anti-Hsp90α, or anti-Hsp90α and rPKM2 together by tail vein injection. **(E)** Representative H&E images of lung metastasis areas. Scale bars, 2 mm. **(F)** The qualification of lung metastasis areas. **(G)** Representative H&E images of liver metastasis areas. Scale bars, 200 μm. **(H)** The qualification of liver metastasis areas. Scale bars, 200 μm. Data are represented as mean ± SEM. NS, not significant; *p <0.05 and **p < 0.01, longitudinal models for analyzing the repeated measures of tumor volume **(A)** or two-tailed Student’s *t* tests **(B, D, F, H)**.

The biological function of extracellular Hsp90α in tumor progression has been well established in our previous study (29). Regarding this, we speculated that eHsp90α was involved in ePKM2-promoted tumor progression *in vivo*. To address this, a xenograft tumor model was established by subcutaneously transplanting A549 cancer cells into nude mice, and the effect of anti-Hsp90α antibody on blocking ePKM2-promoted tumor progression was investigated. Consistent with our previous results, administration of rPKM2 promoted tumor growth (**Figures 4A–D**). Importantly, rPKM2-promoted tumor growth was significantly restrained after the administration of the anti-Hsp90α antibody. Besides, anti-Hsp90α antibody treatment also counteracted rPKM2-induced weight gaining of sentinel lymph nodes (**Figure S3A**).

To exclude the influence of primary tumors on metastasis in our subcutaneous xenograft model, we established another mouse model by *i.v.* injection of A549 cells. Consistently, A549 cells treated with rPKM2 showed elevated lung and liver metastasis (**Figures 4E–H**). Meanwhile, administration of anti-Hsp90α antibody effectively counteracted rPKM2-promoted metastasis, which is consistent with the *in vitro* results (**Figures 4E–H**). In addition, immunohistochemistry (IHC) results showed that the amount of PKM2 in lung tissue was significantly decreased after the treatment with anti-Hsp90α antibody (**Figure S3B**). Furthermore, rPKM2 treatment increased the weight of sentinel lymph nodes. However, rPKM2-increased sentinel lymph nodes weight was significantly attenuated with the treatment with the Hsp90α antibody. (**Figure S3C**). Therefore, we demonstrated that eHsp90α was critical for ePKM2-promoted tumor progression *in vivo*.

### eHsp90α Prevents the Degradation of ePKM2

**Figure 5:**
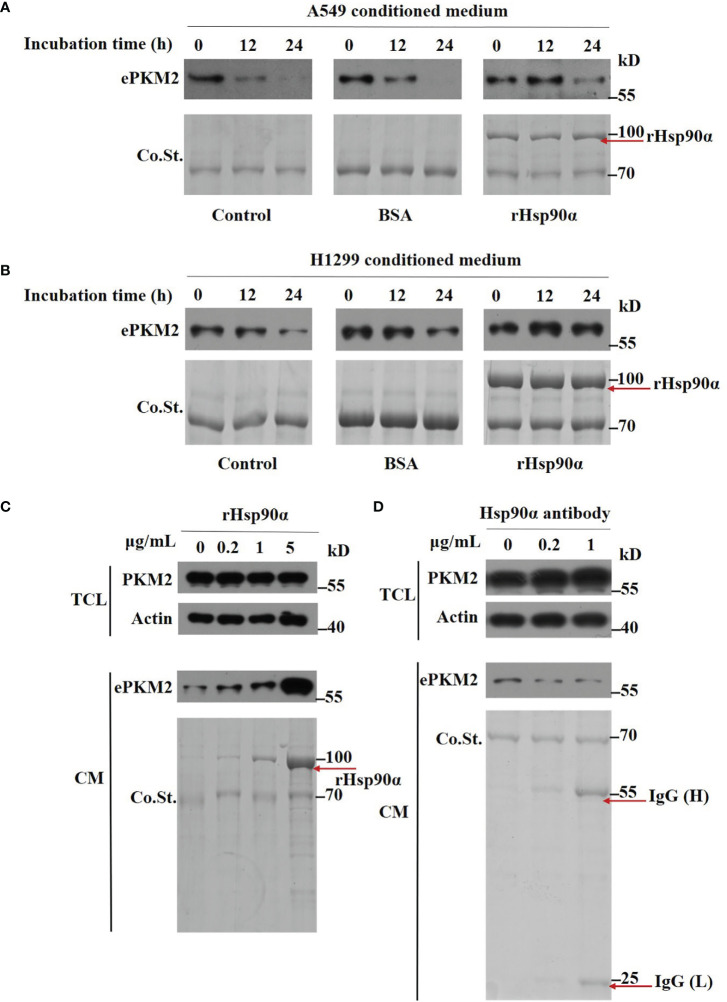
Extracellular Hsp90α stabilizes ePKM2. **(A)** The stabilization of extracellular PKM2 and Hsp90α in A549-conditioned media. PKM2 in A549 CM was examined upon the treatment of BSA or rHsp90α (1 μg/ml) for 12 and 24 h by Western blotting. **(B)** The stabilization of extracellular PKM2 and Hsp90α in H1299-conditioned media. PKM2 in H1299 CM was examined upon the treatment of BSA or rHsp90α (1 μg/ml) for 12 and 24 h by Western blotting. **(C)** Intracellular and extracellular PKM2 in A549 cells were examined upon the treatment of rHsp90α with different concentrations on cells for 12 h by Western blotting. **(D)** Intracellular and extracellular PKM2 in A549 cells were examined upon the treatment of Hsp90α antibody with different concentrations on cells for 12 h by Western blotting.

Next, we wonder how eHsp90α supports ePKM2 to exert its pro-metastasis function. To investigate this, we first detected the amount of PKM2 in conditioned media of A549 cells. We found that while the concentration of PKM2 in conditioned media was significantly decreased after 12 h of culture, Hsp90α was still stable (**Figure S4A**). Furthermore, we found that the degradation of PKM2 was efficiently blocked in the presence of rHsp90, as revealed by half-maintaining of PKM2 after 24 h of culture (**Figure 5A**). Similar results were observed in H1299-conditioned medium (**Figure 5B**). As such, eHsp90α was effective in preventing the degradation of extracellular PKM2 *in vitro*.

To further verify the stabilizing activity of Hsp90α on ePKM2, we treated A549 cells with various doses of rHsp90α or anti-Hsp90α antibody, and the amount of ePKM2 in different conditioned media was measured as above. We found that the level of PKM2 was elevated in correlation with the concentration of rHsp90α (**Figure 5C**). In contrast, anti-Hsp90α antibody treatment exhibited the opposite effect (**Figure 5D**). However, the level of PKM2 in total cell lysates shows little difference among groups (**Figures 5C, D**). Similarly, the level of PKM2 in H1299 cell-conditioned media was also elevated in correlation with the increase of rHsp90α (**Figure S4B**). Overall, these results show that eHsp90α could stabilize extracellular PKM2 in culture.

### eHsp90α Boosts the ePKM2-GRP78-AKT Pathway

**Figure 6:**
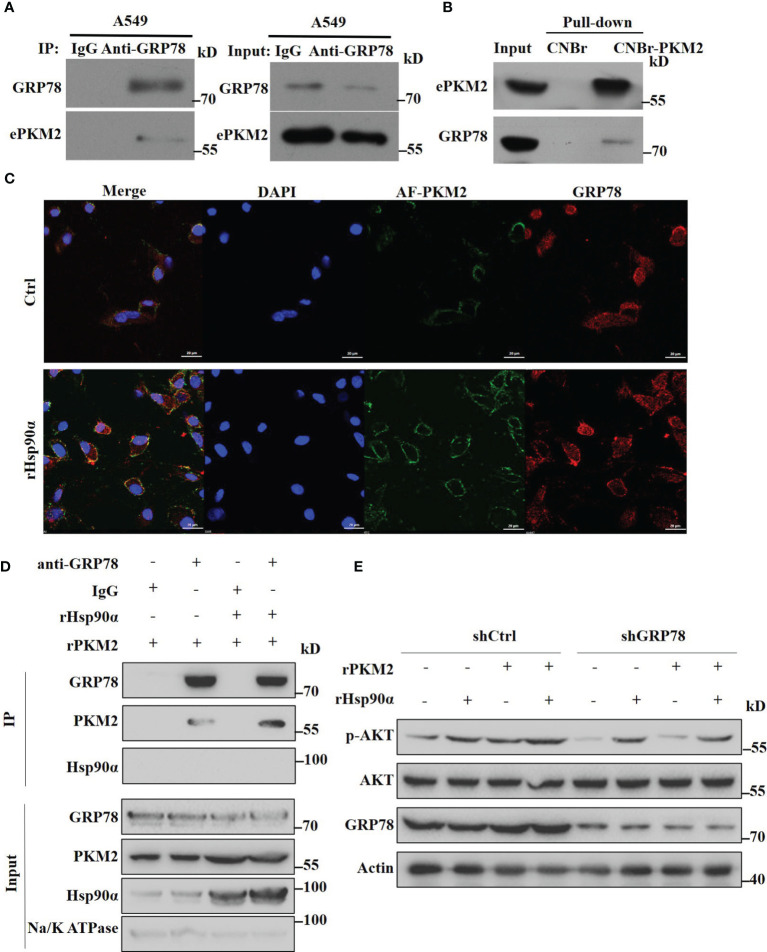
Extracellular Hsp90α boosts the ePKM2-GRP78-AKT pathway. **(A)** A549-conditioned media was collected and immunoprecipitated with IgG or anti-GRP78 antibodies. The immunoprecipitated samples were subjected to WB analysis with the indicated antibodies to detect the interaction between PKM2 and GRP78. **(B)** Pull-down assay showing the interaction between PKM2 and cell surface GRP78. Recombinant PKM2 was coupled to cyanogen bromide-activated Sepharose beads and was incubated with crude fractions of the plasma membrane of A549 cells. **(C)** Co-localization between rPKM2 labeled with Alexa Fluor 488 (AF-PKM2) and GRP78 in A549 cells treated with or without rHsp90α. Scale bar, 20 μm. **(D)** rHsp90α enhances the interaction between rPKM2 and cell surface GRP78. Crude fractions of the plasma membrane of A549 cells were extracted and immunoprecipitated with IgG or anti-GRP78 antibodies treated with rPKM2 and rHsp90α. **(E)** A549-sh Ctrl and A549-sh GRP78 cells were treated with rPKM2 (5 μg/ml), rHsp90α (5 μg/ml), or the combination of Hsp90α and PKM2 for 15 min. Akt phosphorylation levels were detected by western blotting.

Since we have demonstrated that eHsp90α supports ePKM2-promoted tumor metastasis function by stabilizing extracellular PKM2, here we want to investigate the effect of eHSP90α on the downstream signal of ePKM2 on tumor cells. As revealed by mass spectrometry results, GRP78 was also an interacting candidate of ePKM2 in addition to eHsp90α (**Table S1**). Interestingly, it was shown in our previous study that GRP78 is one of the cell membrane-interacting proteins of secreted PKM2 (25). Although GRP78 is usually considered as an intercellular molecular chaperone, it was also reported as the receptor of multiple ligands (31). To explore the interaction between GRP78 and ePKM2, we detected the physical interaction between GRP78 and ePKM2 both in the extracellular environment and on the cell surface, respectively. A549- and H1299-conditioned media were used for anti-GRP78 co-immunoprecipitation analysis. It shows that ePKM2 interacted with GRP78 in the extracellular environment (**Figure 6A** **and** **Figure S5A**). We further investigated the binding affinity of conjugated rPKM2-cyanogen bromide-activated Sepharose beads and crude fractions of the cell membrane of A549 cells. The results showed that GRP78 also interacts with PKM2 on cell membranes (**Figure 6B**).

To confirm this, we treated A549 cells with exogenous Alexa Fluor488-conjugated PKM2 (AF-PKM2) to detect the interaction between ePKM2 and cell surface GRP78. As expected, the co-localization of AF-PKM2 and GRP78 on the A549 cell membrane was demonstrated by confocal immunofluorescence (**Figure 6C**). Besides, treatment with rHsp90α significantly enhanced the colocalization between GRP78 and ePKM2. Furthermore, we treated crude fractions of A549 cell membrane with rPKM2 and rHsp90α to detect the effect of Hsp90α on the interaction between ePKM2 and GRP78. As expected, the interaction between ePKM2 and cell surface GRP78 was dramatically increased by the addition of rHsp90α (**Figure 6D**).

To further verify the role of GRP78 in mediating the signal of ePKM2 on tumor cells, we treated A549 cells with an anti-GRP78 antibody. As shown, anti-GRP78 antibody treatment weakened the binding ability of PKM2 to tumor cells (**Figures S5B**). Since cell surface GRP78 has been reported to activate intracellular AKT signaling to affect tumor cell migration (32), we detected whether ePKM2 can promote GRP78/AKT signaling. It showed that both rHsp90α and rPKM2 can enhance the phosphorylation of AKT (**Figure 6E**). Moreover, coadministration of rHsp90α and rPKM2 elicited a synergistic effect. To investigate the function of GRP78 on ePKM2-induced AKT activation, we knocked down the expression level of GRP78 in A549 cells. It showed that PKM2-stimulated AKT phosphorylation was significantly abrogated in GRP78 knockdown cells. However, little effect was observed on Hsp90α-induced AKT phosphorylation. Therefore, it is demonstrated that eHsp90α boosts the ePKM2-GRP78-AKT pathway by maintaining the stability of ePKM2.

### Targeting GRP78 Suppresses ePKM2-Promoted Tumor Migration and Invasion

**Figure 7:**
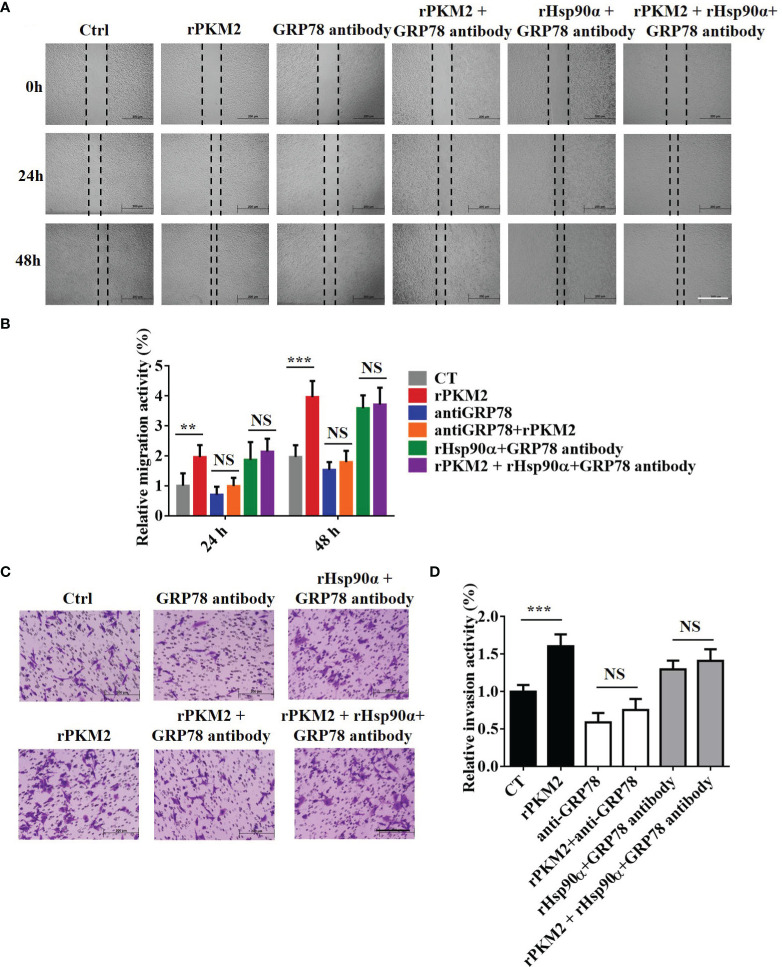
GRP78 mediates secreted PKM2-facilitated tumor migration and invasion. **(A)** Representative images of A549 migration assay (scale bar, 200 μm) with (i) PBS, (ii) rPKM2, (iii) anti-GRP78, (iv) anti-GRP78 and rPKM2 combination, (v) rHsp90α and anti-GRP78 combination, and (vi) rHsp90α, rPKM2, and anti-GRP78 combination. **(B)** Quantified results of A549 migration assay with (i) PBS, (ii) rPKM2, (iii) anti-GRP78, (iv) anti-GRP78 and rPKM2 combination, (v) rHsp90α and anti-GRP78 combination, and (vi) rHsp90α, rPKM2, and anti-GRP78 combination. **(C)** Representative images of A549 invasion assay (scale bar, 200 μm) with (i) PBS, (ii) rPKM2, (iii) anti-GRP78, (iv) anti-GRP78 and rPKM2 combination, (v) rHsp90α and anti-GRP78 combination, and (vi) rHsp90α, rPKM2, and anti-GRP78 combination. **(D)** Quantified results of A549 invasion assay with (i) PBS, (ii) rPKM2, (iii) anti-GRP78, (iv) anti-GRP78 and rPKM2 combination, (v) rHsp90α and anti-GRP78 combination, (vi) rHsp90α, rPKM2, and anti-GRP78 combination. Data are represented as mean ± SD. NS, not significant; **p < 0.01; ***p < 0.001, two-tailed Student’s *t* tests; n = 3 biological replicates.

GRP78 has been shown to be involved in tumor metastasis in previous studies (32). As the role of eHsp90α in supporting ePKM2-GRP78-AKT signaling is revealed, we next want to know whether GRP78 mediates ePKM2-promoted tumor cell migration and invasion. As expected, the results showed that rPKM2-promoted migration and invasion were dramatically inhibited by the treatment of the anti-GRP78 antibody (**Figures 7A–D**), while it cannot be rescued by rHsp90α treatment (**Figures 7A–D**). Moreover, knocking down of GRP78 in A549 cells also weakened the cell invasion prompted by PKM2 (**Figure S6A–C**), which was confirmed by similar observations on H1299 cells (**Figures S6D, E**). The results indicate that targeting GRP78 can suppress ePKM2-promoted tumor migration and invasion. Collectively, extracellular Hsp90α supports the ePKM2-GRP78-AKT axis to promote tumor cell migration and invasion.

## Discussion

Cancer has long been recognized as one of the most fatal diseases worldwide, and lung cancer is one of the most challenging cancer types due to the lack of effective treatments (33). The consistent escalation of cancer occurrences and death globally has prompted us to develop effective treatments. Intracellular proteins are considered potential targets in cancer therapy; however, as many of them are involved in intricate networking (34, 35), drug targeting of intracellular proteins without rational design will inevitably produce numerous side effects as well as limit its curing effectivity. Compared to intracellular proteins, extracellular ones may have fewer side effects due to their simpler interaction network (2). Therefore, specific inhibition of extracellular proteins can be served as an alternative strategy in cancer treatment.

As an aberrant isoform of pyruvate kinase, PKM2 has aroused growing attention due to its important functions in multiple biological processes, particularly in cancer cells. As an evolutionary conserved glycolytic enzyme, PKM2 is primarily localized in the cytosol (36). Importantly, the enzymic activity of PKM2 in converting PEP to pyruvate is considerably low in cancer cells, which leads to the accumulation of metabolic intermediates of glycolysis and eventually promotes the proliferation of cancer cells. Beyond that, PKM2 is also localized in the nucleus, where PKM2 serves as a regulatory component of transcription factors such as HIF-1α (37, 38). Although intracellular PKM2 had been extensively studied, the biological function or the molecular mechanism of extracellular PKM2 in tumor cells is unclear. Our previous study has revealed the role of extracellular PKM2 in facilitating lung cancer progression (25), and the present study which focuses on the regulatory mechanism of ePKM2 will provide new insights on ePKM2-based antitumor therapies.

In this study, we identified the interacting proteins of PKM2 in conditioned media by mass spectrometry analysis. We found that eHsp90α interacts with PKM2 and is involved in ePKM2-promoted tumor progression. Similarly, previous mass spectrometry results also demonstrated that PKM2 was an interacting protein of eHsp90α in MCF-7 (39). Huang et al. reported that HMGCR increases the activity of glycolysis by upregulation of Hsp90 expression, thus maintaining the levels of PKM2 (40). In this study, we first identified the interaction between Hsp90α and PKM2 in the extracellular environment. Hsp90α is an essential and abundant intracellular molecular chaperone that assists in various biological processes and plays significant roles in various biological processes (30, 41–43). Currently, Picard lab had identified nearly 1,000 proteins as intracellular Hsp90 interactors, which have multiple biological functions (http://www.picard.ch/downloads). These substrates and effectors are susceptible to aggregation or degradation without binding to Hsp90α (30, 44). Hsp90α has been found localized on the cell membrane and is secreted into the extracellular environment (6, 7, 44, 45). Usually, eHsp90α executes its functions by interacting with other proteins (46–48). However, studies on the extracellular protein interaction network are limited. Matrix metalloproteinase 9 (MMP9) (49), tissue plasminogen activator (tPA, also known as PLAT) (50), and lysyl oxidase 2-like protein (47) had also been reported to interact with eHsp90α. Accordingly, extracellular Hsp90α could stabilize MMP-2 and protect it from degradation in tumor cells (28, 49, 51). Similarly, we found that eHsp90α can stabilize ePKM2 to enhance tumor-promoting effects caused by ePKM2. Furthermore, anti-Hsp90α antibody treatment weakens ePKM2-promoted tumor metastasis. Therefore, our study enriched the discovery of additional extracellular Hsp90α-interacting proteins, and additional functions of extracellular Hsp90α are revealed as well.

Our previous mass spectrometry results showed that GRP78 was one of the cell membrane-interacting proteins of secreted PKM2, although GRP78 was not recognized to have “receptor activity” according to molecular function analyses (25). Besides its normal functions in the ER, GRP78 could also be translocated to the plasma membrane to activate multiple signals (52). Earlier studies reported that the higher expression of GRP78 was associated with increased malignant behavior of tumor cells (53). Cell surface GRP78 could also serve as a receptor for a large variety of ligands, such as activated α2-macroglobulin (α2M), different types of viruses, plasminogen kringle 5 (K5), microplasminogen, major histocompatibility complex class I (MHC-I), the voltage-dependent anion channel (VDAC), tissue factor (TF), the prostate apoptosis response-4 protein (Par-4), and the teratocarcinoma-derived growth factor 1 (Cripto) (32). Cell surface GPR78 has been reported to activate AKT signaling upon the ligands binding with it. Herein, we verified the interaction between GRP78 and ePKM2 on both extracellular and cell surfaces. In addition, ePKM2 binds to cell surface GRP78 to activate AKT signaling, and eHsp90α enhances the ePKM2-GRP78-AKT axis. Furthermore, the anti-GRP78 antibody blocks ePKM2-promoted tumor migration and invasion. Therefore, our study further confirmed the function of cell-surface GRP78 on mediating ePKM2-promoted tumor metastasis, which uncovered a novel mechanism of extracellular PKM2 in facilitating tumor progression.

In conclusion, our study first shows the extracellular interaction of ePKM2 with eHsp90α and illustrated the mechanism of eHsp90α in regulating ePKM2. Besides, GRP78 is shown to mediate ePKM2-promoted tumor progression. Overall, our study provides novel concepts for those extracellular protein interactions which can be beneficial for future development of lung cancer therapies.

## Data Availability Statement

The original contributions presented in the study are included in the article/**Supplementary Material**. Further inquiries can be directed to the corresponding author.

## Ethics Statement

The animal study was reviewed and approved by Binzhou Medical University.

## Author Contributions

SSZ: experimental operation, writing—original draft preparation; CHW: data curation and investigation, writing—original draft preparation; JJJ: resources, investigation. CXW: writing—reviewing and editing, supervision. All authors contributed to the article and approved the submitted version.

## Funding

This work was supported by Binzhou Medical University. This work was funded by the National Natural Science Foundation of China (82103071 and 42106152), Shandong Provincial Natural Science Foundation (ZR2021MD087) and Scientific Research Foundation of Binzhou Medical University (BY2020KYQD15).

## Conflict of Interest

The authors declare that the research was conducted in the absence of any commercial or financial relationships that could be construed as a potential conflict of interest.

## Publisher’s Note

All claims expressed in this article are solely those of the authors and do not necessarily represent those of their affiliated organizations, or those of the publisher, the editors and the reviewers. Any product that may be evaluated in this article, or claim that may be made by its manufacturer, is not guaranteed or endorsed by the publisher.

## References

[1] da Cunha BR Domingos C Stefanini ACB Henrique T Polachini GM Castelo-Branco P. Cellular Interactions in the Tumor Microenvironment: The Role of Secretome. J Cancer (2019) 10(19):4574–87. doi: 10.7150/jca.21780 PMC6746126 31528221

[2] Caccia D Zanetti Domingues L Micciche F De Bortoli M Carniti C Mondellini P. Secretome Compartment Is a Valuable Source of Biomarkers for Cancer-Relevant Pathways. J Proteome Res (2011) 10(9):4196–207. doi: 10.1021/pr200344n 21751813

[3] Zhong L Roybal J Chaerkady R Zhang W Choi K Alvarez CA. Identification of Secreted Proteins That Mediate Cell-Cell Interactions in an *In Vitro* Model of the Lung Cancer Microenvironment. Cancer Res (2008) 68(17):7237–45. doi: 10.1158/0008-5472.CAN-08-1529 PMC2562343 18757440

[4] Kessenbrock K Plaks V Werb Z. Matrix Metalloproteinases: Regulators of the Tumor Microenvironment. Cell (2010) 141(1):52–67. doi: 10.1016/j.cell.2010.03.015 20371345 PMC2862057

[5] Carmeliet P. VEGF as a Key Mediator of Angiogenesis in Cancer. Oncology (2005) 69 Suppl 3:4–10. doi: 10.1159/000088478 16301830

[6] Eustace BK Sakurai T Stewart JK Yimlamai D Unger C Zehetmeier C. Functional Proteomic Screens Reveal an Essential Extracellular Role for Hsp90 Alpha in Cancer Cell Invasiveness. Nat Cell Biol (2004) 6(6):507–14. doi: 10.1038/ncb1131 15146192

[7] Li W Li Y Guan S Fan J Cheng CF Bright AM. Extracellular Heat Shock Protein-90alpha: Linking Hypoxia to Skin Cell Motility and Wound Healing. EMBO J (2007) 26(5):1221–33. doi: 10.1038/sj.emboj.7601579 PMC1817627 17304217

[8] Strandholm JJ Dyson RD Cardenas JM. Bovine Pyruvate Kinase Isozymes and Hybrid Isozymes. Electrophoretic Studies and Tissue Distribution. Arch Biochem Biophys (1976) 173(1):125–31. doi: 10.1016/0003-9861(76)90242-3 1259434

[9] Christofk HR Vander Heiden MG Harris MH Ramanathan A Gerszten RE Wei R. The M2 Splice Isoform of Pyruvate Kinase Is Important for Cancer Metabolism and Tumour Growth. Nature (2008) 452(7184):230–3. doi: 10.1038/nature06734 18337823

[10] Vander Heiden MG Cantley LC Thompson CB. Understanding the Warburg Effect: The Metabolic Requirements of Cell Proliferation. Science (2009) 324(5930):1029–33. doi: 10.1126/science.1160809 PMC2849637 19460998

[11] Mazurek S Boschek CB Hugo F Eigenbrodt E. Pyruvate Kinase Type M2 and its Role in Tumor Growth and Spreading. Semin Cancer Biol (2005) 15(4):300–8. doi: 10.1016/j.semcancer.2005.04.009 15908230

[12] Huang P Zhao X Xiao W Dong Y Hu G. 18f-Fluorodeoxyglucose Uptake Predicts PKM2 Expression in Lung Adenocarcinoma. Oncotarget (2017) 8(24):39618–26. doi: 10.18632/oncotarget.17377 PMC5503637 28489603

[13] Sun T Liu Z Bi F Yang Q. Deubiquitinase PSMD14 Promotes Ovarian Cancer Progression by Decreasing Enzymatic Activity of PKM2. Mol Oncol (2021) 15(12):3639–58. doi: 10.1002/1878-0261.13076 PMC8637564 34382324

[14] Liang J Cao R Wang X Zhang Y Wang P Gao H. Mitochondrial PKM2 Regulates Oxidative Stress-Induced Apoptosis by Stabilizing Bcl2. Cell Res (2017) 27(3):329–51. doi: 10.1038/cr.2016.159 PMC5339831 28035139

[15] Jiang Y Wang Y Wang T Hawke DH Zheng Y Li X. PKM2 Phosphorylates MLC2 and Regulates Cytokinesis of Tumour Cells. Nat Commun (2014) 5:5566. doi: 10.1038/ncomms6566 25412762 PMC4259466

[16] Yang W Xia Y Hawke D Li X Liang J Xing D. PKM2 Phosphorylates Histone H3 and Promotes Gene Transcription and Tumorigenesis. Cell (2014) 158(5):1210. doi: 10.1016/j.cell.2012.07.018 28917293

[17] Gao X Wang H Yang JJ Liu X Liu ZR. Pyruvate Kinase M2 Regulates Gene Transcription by Acting as a Protein Kinase. Mol Cell (2012) 45(5):598–609. doi: 10.1016/j.molcel.2012.01.001 22306293 PMC3299833

[18] Yang W Xia Y Ji H Zheng Y Liang J Huang W. Nuclear PKM2 Regulates Beta-Catenin Transactivation Upon EGFR Activation. Nature (2011) 480(7375):118–22. doi: 10.1038/nature10598 PMC3235705 22056988

[19] Azoitei N Becher A Steinestel K Rouhi A Diepold K Genze F. PKM2 Promotes Tumor Angiogenesis by Regulating HIF-1alpha Through NF-kappaB Activation. Mol Cancer (2016) 15:3. doi: 10.1186/s12943-015-0490-2 26739387 PMC4704385

[20] Amin S Yang P Li Z. Pyruvate Kinase M2: A Multifarious Enzyme in non-Canonical Localization to Promote Cancer Progression. Biochim Biophys Acta Rev Cancer (2019) 1871(2):331–41. doi: 10.1016/j.bbcan.2019.02.003 30826427

[21] Li L Zhang Y Qiao J Yang JJ Liu ZR. Pyruvate Kinase M2 in Blood Circulation Facilitates Tumor Growth by Promoting Angiogenesis. J Biol Chem (2014) 289(37):25812–21. doi: 10.1074/jbc.M114.576934 PMC4162182 25070887

[22] Kim H Kim SH Hwang D An J Chung HS Yang EG. Extracellular Pyruvate Kinase M2 Facilitates Cell Migration by Upregulating Claudin-1 Expression in Colon Cancer Cells. Biochem Cell Biol (2020) 98(2):219–26. doi: 10.1139/bcb-2019-0139 31545907

[23] Yang P Li Z Wang Y Zhang L Wu H Li Z. Secreted Pyruvate Kinase M2 Facilitates Cell Migration *via* PI3K/Akt and Wnt/beta-Catenin Pathway in Colon Cancer Cells. Biochem Biophys Res Commun (2015) 459(2):327–32. doi: 10.1016/j.bbrc.2015.02.112 25732087

[24] Han H Zhang Y Peng G Li L Yang J Yuan Y. Extracellular PKM2 Facilitates Organ-Tissue Fibrosis Progression. iScience (2021) 24(10):103165. doi: 10.1016/j.isci.2021.103165 34693222 PMC8517170

[25] Wang C Zhang S Liu J Tian Y Ma B Xu S. Secreted Pyruvate Kinase M2 Promotes Lung Cancer Metastasis Through Activating the Integrin Beta1/FAK Signaling Pathway. Cell Rep (2020) 30(6):1780–97. doi: 10.1016/j.celrep.2020.01.037 32049010

[26] Wang D Zhao C Xu F Zhang A Jin M Zhang K. Cisplatin-Resistant NSCLC Cells Induced by Hypoxia Transmit Resistance to Sensitive Cells Through Exosomal Pkm2. Theranostics (2021) 11(6):2860–75. doi: 10.7150/thno.51797 PMC7806469 33456577

[27] Hou PP Luo LJ Chen HZ Chen QT Bian XL Wu SF. Ectosomal PKM2 Promotes HCC by Inducing Macrophage Differentiation and Remodeling the Tumor Microenvironment. Mol Cell (2020) 78(6):1192–206. doi: 10.1016/j.molcel.2020.05.004 32470318

[28] Song X Wang X Zhuo W Shi H Feng D Sun Y. The Regulatory Mechanism of Extracellular Hsp90{alpha} on Matrix Metalloproteinase-2 Processing and Tumor Angiogenesis. J Biol Chem (2010) 285(51):40039–49. doi: 10.1074/jbc.M110.181941 PMC3000986 20937816

[29] Zhang S Wang C Ma B Xu M Xu S Liu J. Mutant P53 Drives Cancer Metastasis *via* RCP-Mediated Hsp90alpha Secretion. Cell Rep (2020) 32(1):107879. doi: 10.1016/j.celrep.2020.107879 32640214

[30] Whitesell L Lindquist SL. HSP90 and the Chaperoning of Cancer. Nat Rev Cancer (2005) 5(10):761–72. doi: 10.1038/nrc1716 16175177

[31] Ni M Zhang Y Lee AS. Beyond the Endoplasmic Reticulum: Atypical GRP78 in Cell Viability, Signalling and Therapeutic Targeting. Biochem J (2011) 434(2):181–8. doi: 10.1042/BJ20101569 PMC3353658 21309747

[32] Gonzalez-Gronow M Gopal U Austin RC Pizzo SV. Glucose-Regulated Protein (GRP78) Is an Important Cell Surface Receptor for Viral Invasion, Cancers, and Neurological Disorders. IUBMB Life (2021) 73(6):843–54. doi: 10.1002/iub.2502 33960608

[33] Sung H Ferlay J Siegel RL Laversanne M Soerjomataram I Jemal A. Global Cancer Statistics 2020: GLOBOCAN Estimates of Incidence and Mortality Worldwide for 36 Cancers in 185 Countries. CA Cancer J Clin (2021) 71(3):209–49. doi: 10.3322/caac.21660 33538338

[34] Obenauf AC Zou Y Ji AL Vanharanta S Shu W Shi H. Therapy-Induced Tumour Secretomes Promote Resistance and Tumour Progression. Nature (2015) 520(7547):368–72. doi: 10.1038/nature14336 PMC4507807 25807485

[35] Stelzl U Worm U Lalowski M Haenig C Brembeck FH Goehler H. A Human Protein-Protein Interaction Network: A Resource for Annotating the Proteome. Cell (2005) 122(6):957–68. doi: 10.1016/j.cell.2005.08.029 16169070

[36] Clower CV Chatterjee D Wang Z Cantley LC Vander Heiden MG Krainer AR. The Alternative Splicing Repressors hnRNP A1/A2 and PTB Influence Pyruvate Kinase Isoform Expression and Cell Metabolism. Proc Natl Acad Sci U S A (2010) 107(5):1894–9. doi: 10.1073/pnas.0914845107 PMC2838216 20133837

[37] Luo W Hu H Chang R Zhong J Knabel M O'Meally R. Pyruvate Kinase M2 Is a PHD3-Stimulated Coactivator for Hypoxia-Inducible Factor 1. Cell (2011) 145(5):732–44. doi: 10.1016/j.cell.2011.03.054 PMC3130564 21620138

[38] Wang HJ Hsieh YJ Cheng WC Lin CP Lin YS Yang SF. JMJD5 Regulates PKM2 Nuclear Translocation and Reprograms HIF-1alpha-Mediated Glucose Metabolism. Proc Natl Acad Sci U.S.A. (2014) 111(1):279–84. doi: 10.1073/pnas.1311249111 PMC3890888 24344305

[39] Tian Y Wang C Chen S Liu J Fu Y Luo Y. Extracellular Hsp90alpha and Clusterin Synergistically Promote Breast Cancer Epithelial-To-Mesenchymal Transition and Metastasis *via* LRP1. J Cell Sci (2019) 132(15):jcs228213. doi: 10.1242/jcs.228213 31273033

[40] Huang J Zhao X Li X Peng J Yang W Mi S. HMGCR Inhibition Stabilizes the Glycolytic Enzyme PKM2 to Support the Growth of Renal Cell Carcinoma. PLoS Biol (2021) 19(4):e3001197. doi: 10.1371/journal.pbio.3001197 33905408 PMC8104400

[41] Trepel J Mollapour M Giaccone G Neckers L. Targeting the Dynamic HSP90 Complex in Cancer. Nat Rev Cancer (2010) 10(8):537–49. doi: 10.1038/nrc2887 PMC6778733 20651736

[42] Woodford MR Backe SJ Wengert LA Dunn DM Bourboulia D Mollapour M. Hsp90 Chaperone Code and the Tumor Suppressor VHL Cooperatively Regulate the Mitotic Checkpoint. Cell Stress Chaperones (2021) 26(6):965–71. doi: 10.1007/s12192-021-01240-2 PMC8578495 34586601

[43] Backe SJ Sager RA Woodford MR Makedon AM Mollapour M. Post-Translational Modifications of Hsp90 and Translating the Chaperone Code. J Biol Chem (2020) 295(32):11099–117. doi: 10.1074/jbc.REV120.011833 PMC7415980 32527727

[44] Caplan AJ Mandal AK Theodoraki MA. Molecular Chaperones and Protein Kinase Quality Control. Trends Cell Biol (2007) 17(2):87–92. doi: 10.1016/j.tcb.2006.12.002 17184992

[45] Cortes S Baker-Williams AJ Mollapour M Bourboulia D. Detection and Analysis of Extracellular Hsp90 (Ehsp90). Methods Mol Biol (2018) 1709:321–9. doi: 10.1007/978-1-4939-7477-1_23 29177669

[46] Wong DS Jay DG. Emerging Roles of Extracellular Hsp90 in Cancer. Adv Cancer Res (2016) 129:141–63. doi: 10.1016/bs.acr.2016.01.001 26916004

[47] McCready J Wong DS Burlison JA Ying W Jay DG. An Impermeant Ganetespib Analog Inhibits Extracellular Hsp90-Mediated Cancer Cell Migration That Involves Lysyl Oxidase 2-Like Protein. Cancers (Basel) (2014) 6(2):1031–46. doi: 10.3390/cancers6021031 PMC4074815 24785146

[48] Eustace BK Jay DG. Extracellular Roles for the Molecular Chaperone, Hsp90. Cell Cycle (2004) 3(9):1098–100. doi: 10.4161/cc.3.9.1088 15326368

[49] Stellas D El Hamidieh A Patsavoudi E. Monoclonal Antibody 4c5 Prevents Activation of MMP2 and MMP9 by Disrupting Their Interaction With Extracellular HSP90 and Inhibits Formation of Metastatic Breast Cancer Cell Deposits. BMC Cell Biol (2010) 11:51. doi: 10.1186/1471-2121-11-51 20602761 PMC2914660

[50] McCready J Sims JD Chan D Jay DG. Secretion of Extracellular Hsp90alpha *via* Exosomes Increases Cancer Cell Motility: A Role for Plasminogen Activation. BMC Cancer (2010) 10:294. doi: 10.1186/1471-2407-10-294 20553606 PMC3087318

[51] Baker-Williams AJ Hashmi F Budzynski MA Woodford MR Gleicher S Himanen SV. Co-Chaperones TIMP2 and AHA1 Competitively Regulate Extracellular HSP90:Client MMP2 Activity and Matrix Proteolysis. Cell Rep (2019) 28(7):1894–906. doi: 10.1016/j.celrep.2019.07.045 PMC8276117 31412254

[52] Tsai YL Zhang Y Tseng CC Stanciauskas R Pinaud F Lee AS. Characterization and Mechanism of Stress-Induced Translocation of 78-Kilodalton Glucose-Regulated Protein (GRP78) to the Cell Surface. J Biol Chem (2015) 290(13):8049–64. doi: 10.1074/jbc.M114.618736 PMC4375463 25673690

[53] Lee AS. GRP78 Induction in Cancer: Therapeutic and Prognostic Implications. Cancer Res (2007) 67(8):3496–9. doi: 10.1158/0008-5472.CAN-07-0325 17440054

