# Supplementary material for: Extracellular Hsp90α Supports the ePKM2-GRP78-AKT Axis to Promote Tumor Metastasis
Source: Front Oncol. 2022 Jun 30;12:906080. doi: 10.3389/fonc.2022.906080 (PMC9280132; doi:10.3389/fonc.2022.906080)
Supplement: Supplementary file 1 [file DataSheet_1.docx]

Supplementary Material

# Supplementary Figures and Tables

## Supplementary Figures


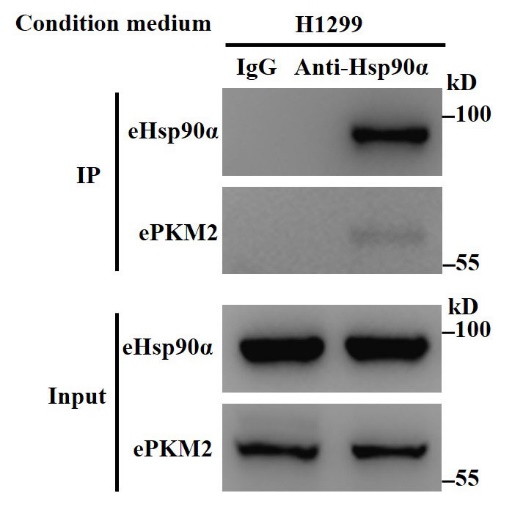


**Supplementary Figure 1.** **eHsp90α can interact with extracellular PKM2.** H1299 cells were lysed and immunoprecipitated with IgG or Hsp90α antibodies. The immunoprecipitated samples were subjected to WB analysis with the indicated antibodies to detect the interaction between extracellular PKM2 and Hsp90α.


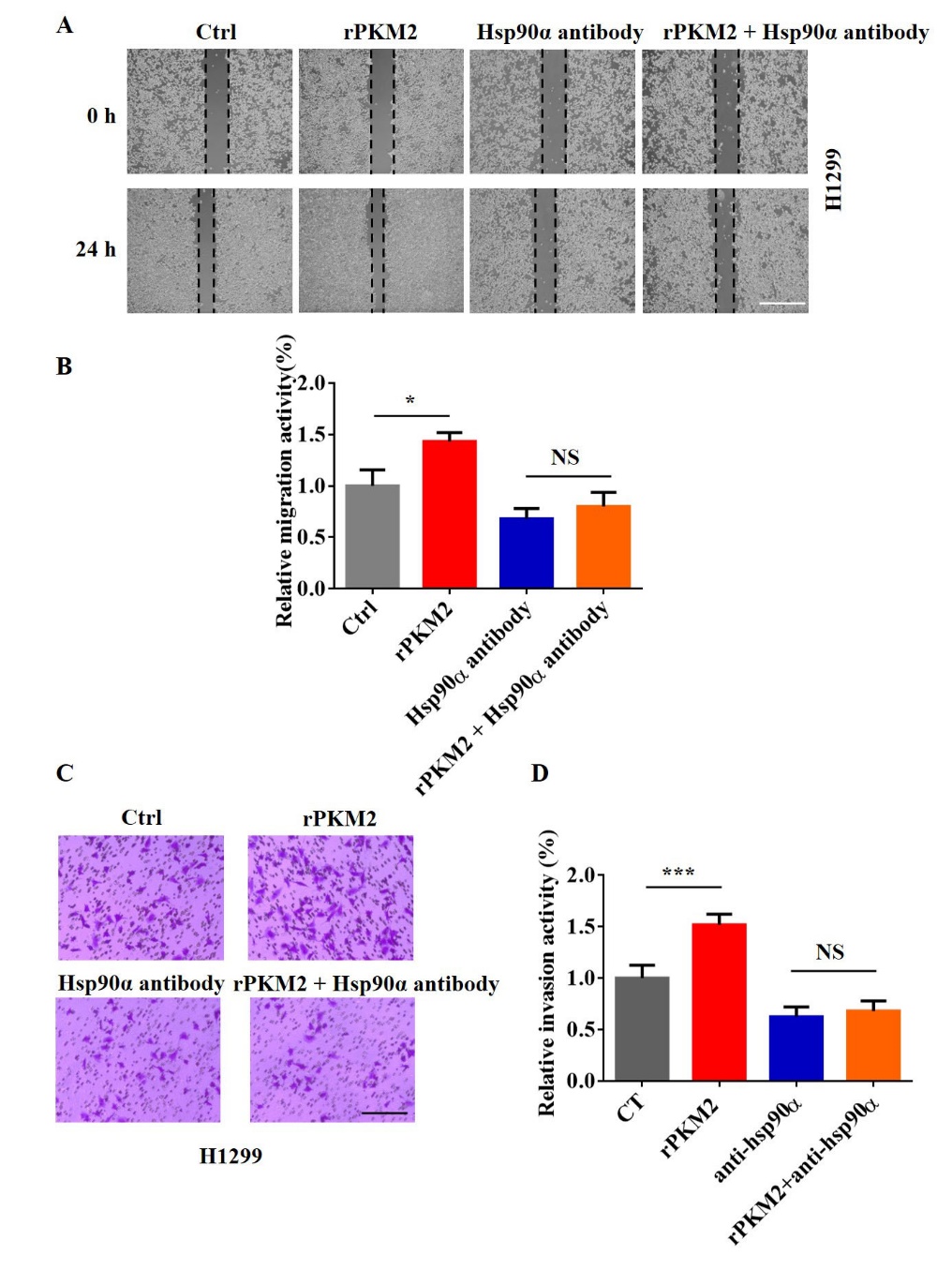


**Supplementary Figure 2. Extracellular PKM2 promotes H1299 cell migration and invasion dependent on extracellular Hsp90α.** A. Representative images and B. quantified results of H1299 migration assay (scale bar, 200 μm) with PBS, rPKM2, anti-Hsp90α, or anti-Hsp90α and rPKM2 together. C. Representative images and D. quantified results of H1299 invasion assay (scale bar, 200 μm) with PBS, rPKM2, anti-Hsp90α, or anti-Hsp90α and rPKM2 together. Data are represented as mean ± SD. NS, not significant; **p* < 0.05, ****p* < 0.001, two-tailed Student’s t tests; n = 3 biological replicates.


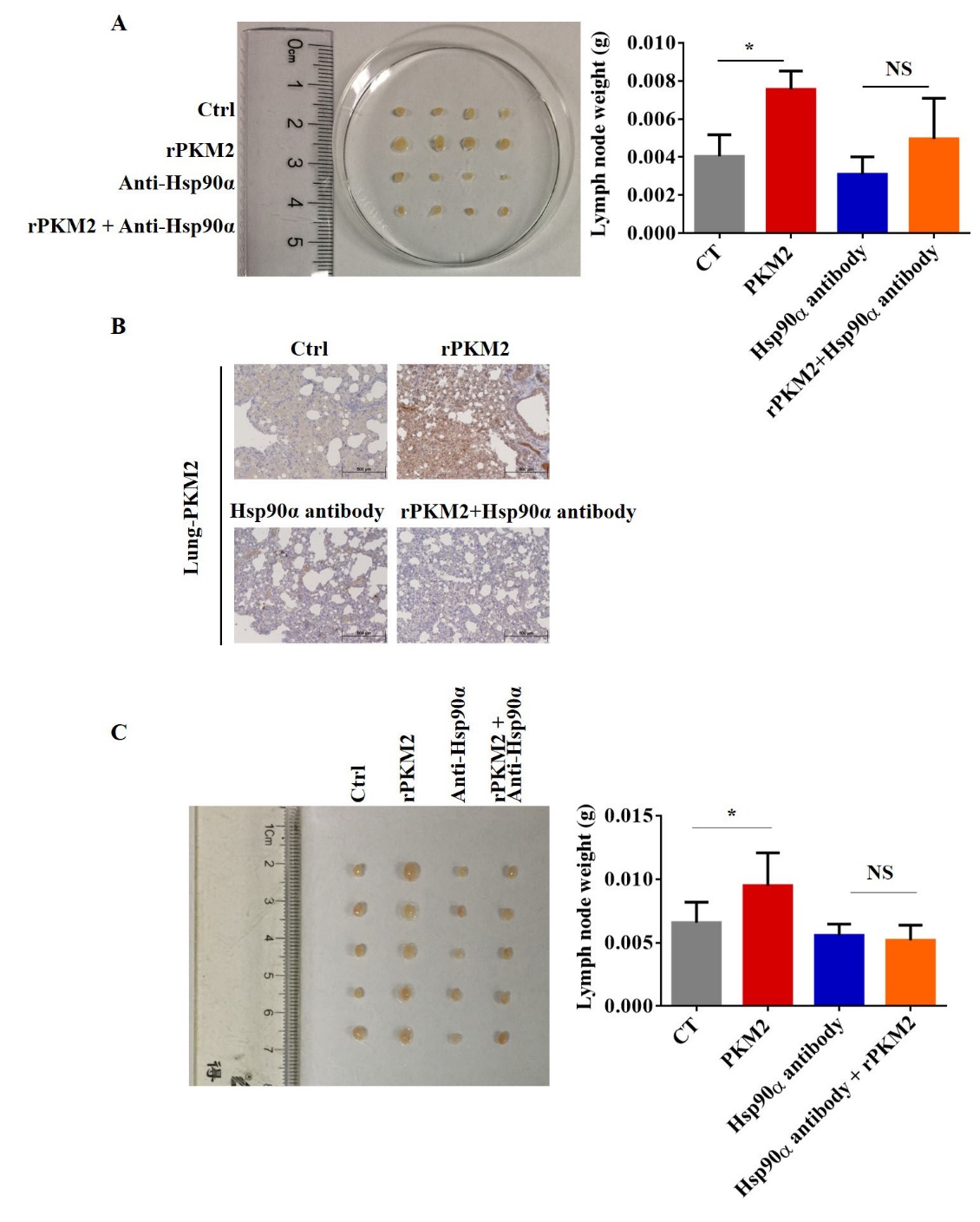


**Supplementary Figure 3. Extracellular PKM2 promotes tumor progression dependent on extracellular** **Hsp90α.** A Tumor progression generated by subcutaneous injection of A549 cells into nude mice (n = 7-8/group). Mice were treated twice per week with PBS, r-PKM2, anti-Hsp90α, or anti-Hsp90α and r-PKM2 together by tail vein injection. Left panels show the gross observation of lymph nodes. Right panels show the weights of lymph nodes in A549 tumor xenografts. B-C. Tumor metastasis generated by tail vein injection of A549 cells into nude mice (n = 7-8/group). Mice were treated twice per week with PBS, r-PKM2, anti-Hsp90α, or anti-Hsp90α and r-PKM2 together by tail vein injection.(B) The qualification of PKM2 of lung tissues in A549 tumor xenografts. Scale bar, 500 μm.(C) Left panels show the gross observation of lymph nodes. Right panels show the weights of lymph nodes. Data are represented as mean ± SEM. NS, not significant; **p* < 0.05, two-tailed Student’s t tests.


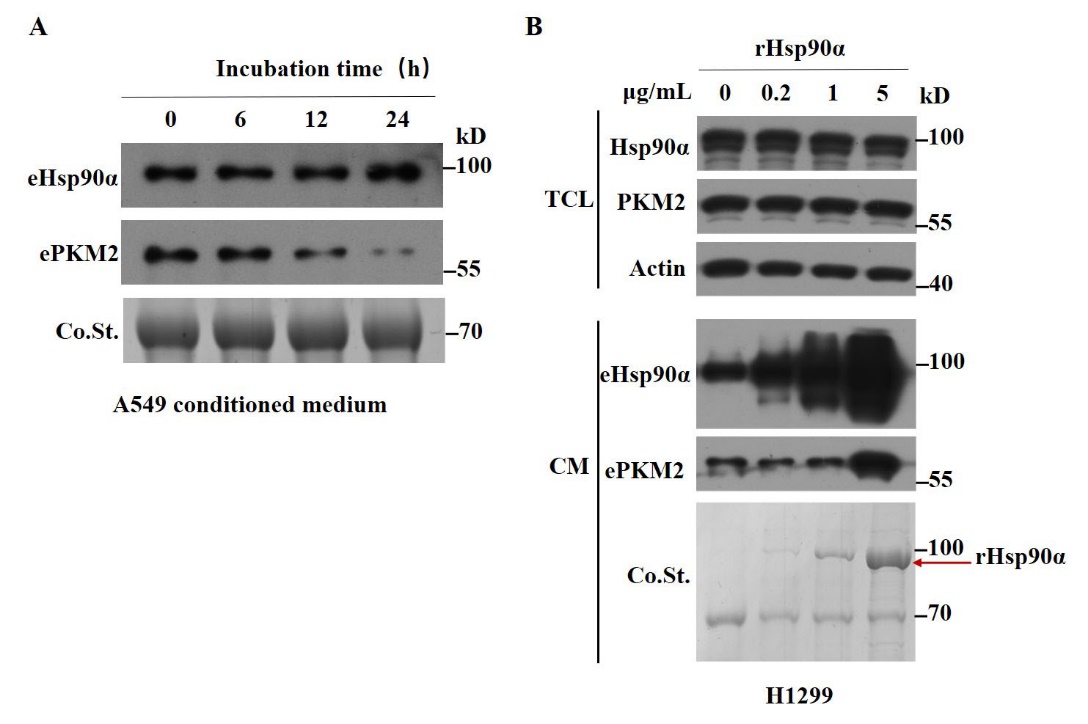


**Supplementary Figure 4. The stabilization of extracellular PKM2 and Hsp90α.** A. A549 conditioned medium was collected. Hsp90α and PKM2 were detected by western blotting after different times. B. Intracellular and extracellular PKM2 in H1299 cells were examined upon the treatment of rHsp90α with different concentrations on cells for 12 h by Western blotting.


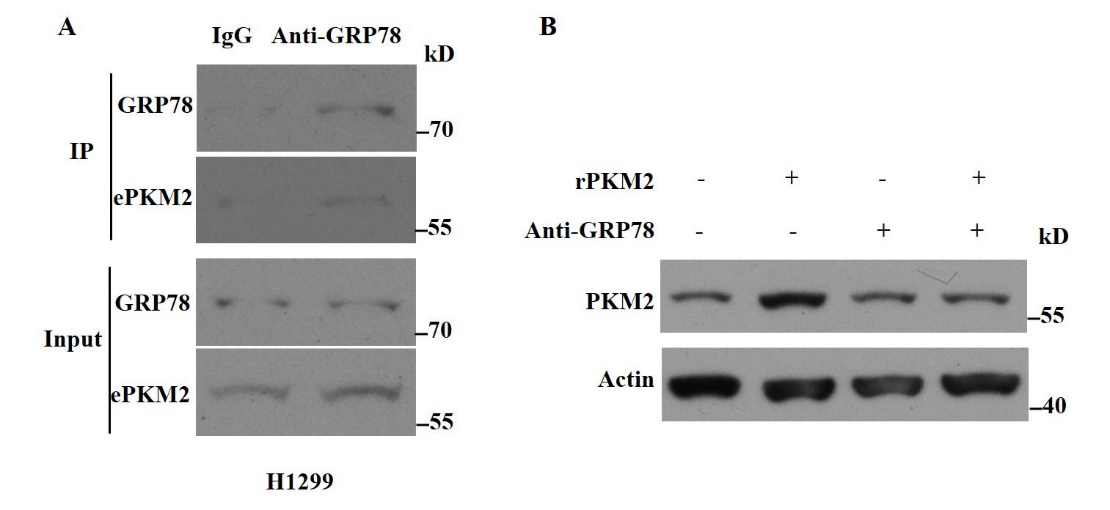


**Supplementary Figure 5. The roles of GRP78 on PKM2 binding to cells.** A. H1299 conditioned media was collected and immunoprecipitated with IgG or anti-GRP78 antibodies. The immunoprecipitated samples were subjected to WB analysis with the indicated antibodies to detect the interaction between PKM2 and GRP78. B. Blocking GRP78 with respective antibody and detecting binding of r-PKM2 to cells in A549 cells using WB.


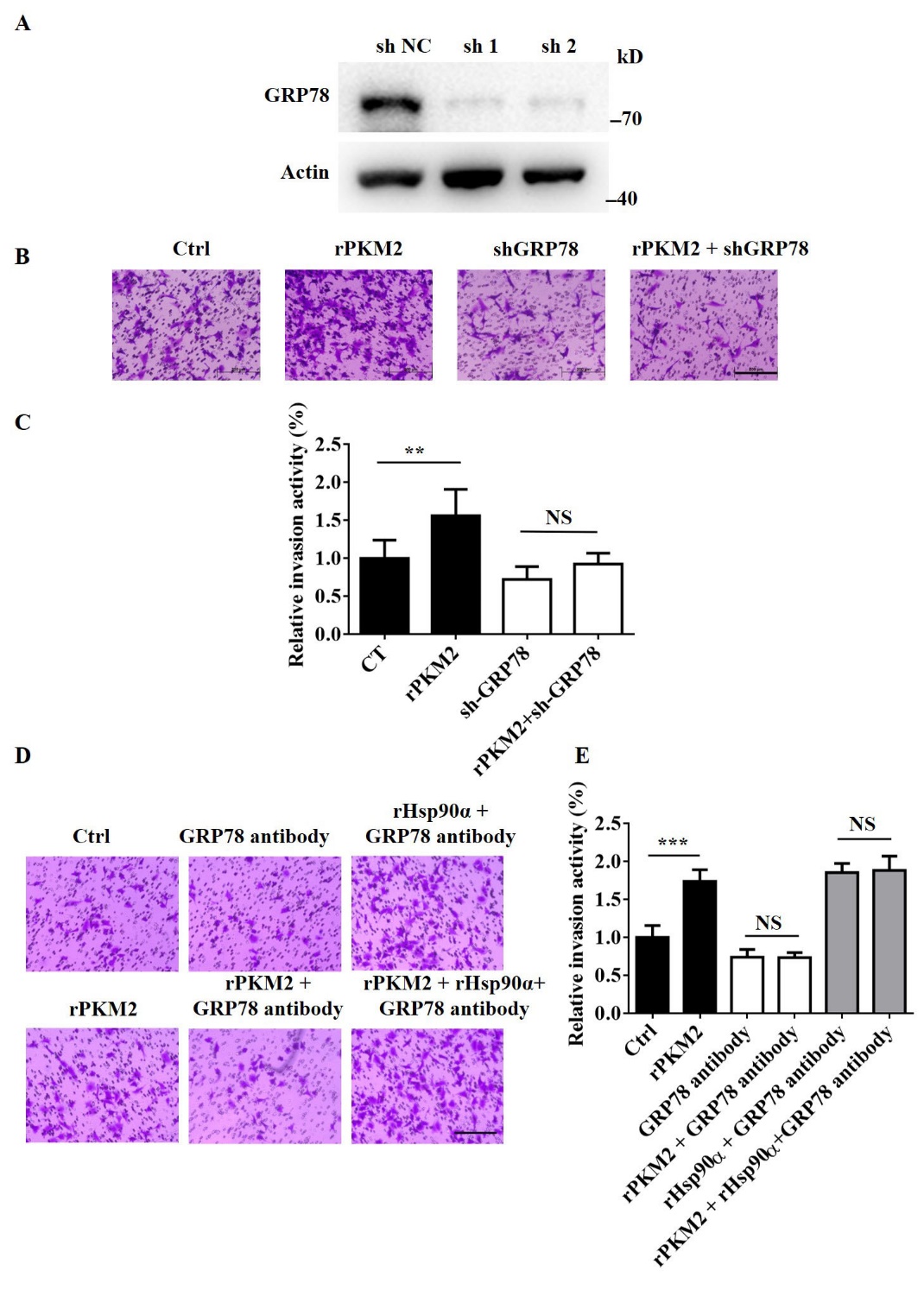


**Supplementary Figure 6. GRP78 Mediates PKM2-facilitated tumor cell invasion.** A. GRP78 knockdown in A549 cells detected using WB. B. Representative images and C. quantified results of A549 invasion assay in WT and GRP78-knockdown A549 cells treated with or without r-PKM2. D. Representative images and E. quantified results of H1299 invasion assay (scale bar, 200 μm) with (i) PBS, (ii) rPKM2, (iii) anti-GRP78, (iv) anti-GRP78 and rPKM2 combination, (v) rHsp90α and anti-GRP78 combination, (vi) rHsp90α, rPKM2 and anti-GRP78 combination. Scale bar, 200 μm. Data are represented as mean ± SD. NS, not significant; ***p* < 0.01; ****p* < 0.001, two-tailed Student’s t tests; n = 3 biological replicates.

## Supplementary Tables

**Supplementary Table 1. The top ten interacting proteins of PKM2 in A549 conditioned medium**

| Accession | Description | Score | # Proteins |
| --- | --- | --- | --- |
| P14618 | Pyruvate kinase PKM | 7123.66 | 24 |
| P07900 | Heat shock protein HSP 90-alpha | 378.72 | 13 |
| P07355 | Annexin A2 | 317.60 | 27 |
| Q9NZM1 | Myoferlin | 306.49 | 4 |
| P11021 | 78 kDa glucose-regulated protein | 306.18 | 2 |
| P08238 | Heat shock protein HSP 90-beta | 229.43 | 12 |
| F5GZS6 | 4F2 cell-surface antigen heavy chain | 202.78 | 12 |
| Q96HX3 | Dolichyl-diphosphooligosaccharide--protein glycosyltransferase subunit 1 | 198.67 | 8 |
| P11142 | Heat shock cognate 71 kDa protein | 192.54 | 26 |
| Q14697 | Neutral alpha-glucosidase AB | 191.75 | 7 |
| P22695 | Cytochrome b-c1 complex subunit 2 | 171.12 | 7 |

**Supplementary Table 2. shRNAs sequences**

| Description | Oligonucleotides |
| --- | --- |
| Human GRP78-shRNA#1 | CCGGCTTGTTGGTGGCTCGACTCGACTCGAGTCGAGTCGAGCCACCAACAAGTTTTT |
| Human GRP78-shRNA#2 | GTACCGGAGATTCAGCAACTGGTTAAAGCTCGAGCTTTAACCAGTTGCTGAATCTTTTTTTG |
